# Women in chemistry: Q&A with Professor Mónica H. Pérez-Temprano

**DOI:** 10.1038/s42004-025-01431-3

**Published:** 2025-02-12

## Abstract

Professor Mónica H. Pérez-Temprano is a professor in Organic Chemistry focused on mechanism-driven reaction design & development.

**Figure:**
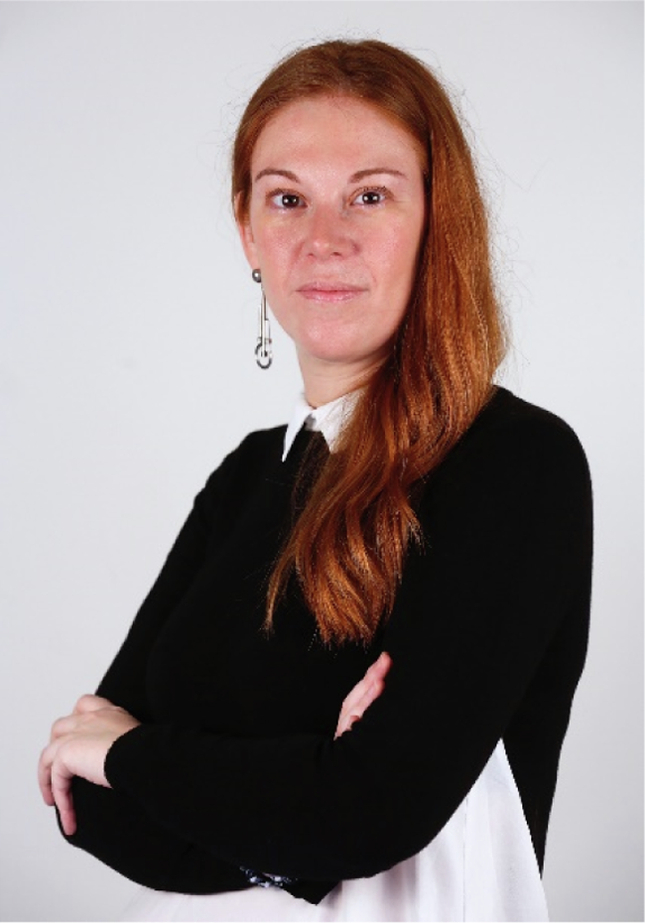
Mónica H. Pérez-Temprano

Mónica H. Pérez-Temprano obtained her BS degree in Chemistry at the University of Valladolid in 2005, and her PhD in 2011 under the supervision of Prof. Espinet and Prof. Casares. As a graduate student, she investigated the mechanisms of different palladium-catalyzed processes. Next, she joined the research group of Prof. Melanie Sanford at the University of Michigan as Ramón Areces fellow, where she focused on the synthesis and reactivity of high-valent palladium(IV) complexes. In 2015, she began her independent career, first as Junior Group Leader at the Institute of Chemical Research of Catalonia (ICIQ) and in 2022, she consolidated her position as Senior Group Leader.

At ICIQ, the Pérez-Temprano group is focused on using mechanisms as a priori tool for developing innovative and more sustainable first-row metal-catalyzed transformations. Her research career has been recognized with different awards and honors including her selection as one of the “Talented 12” of 2018 by *Chemical & Engineering News* (C&EN), the 2020 Young Investigator Group Leader Award by the Spanish Royal Society of Chemistry or the 2021 Young Investigator Award by the EuChemS Division of Organic Chemistry. In addition, she is a member of the International Advisory Board of *Organometallics*, *Chem Catalysis* and the Early Career Advisory Board of *Chemistry – A European Journal*.

Why did you choose to be a scientist?

Each of us has a personal and unique story when it comes to choosing a career, and in my case, I don’t think I *chose* to be a scientist—rather, I believe science chose me. As a female researcher, when I give talks in high schools, many students assume that my inspiration came from someone like Marie Curie. But the truth is, my journey was not so straightforward. For a long time, I didn’t feel like I quite fit in, and science became my safe space—a place where I could explore, question, and belong. From an early age, I was fascinated not just by the names of reactions or compounds, but by the mechanisms behind chemical transformations. I wanted to understand the *why* and the *how* of the chemical processes that shape our world.

As my scientific journey started, I had the privilege of working with supervisors who were not only exceptional researchers but also inspiring mentors and role models. Their guidance and support were instrumental in shaping my path. It was in those moments of discovery and mentorship that I realized my calling: to contribute to advancing chemical reactions and train the next generations of researchers.

What scientific development are you currently most excited about?

I am particularly excited about the opportunities in unlocking the full potential of first-row transition metals in catalysis. In recent years, the global focus toward enhancing the sustainability of chemical processes has fueled renewed interest in incorporating these metals into the organometallic toolkit. However, still now their underutilization remains a long-standing challenge among practitioners of organometallic catalysis due to their perceived erratic behavior. One approach to overcome this limitation is to explore their ability to mimic the well-established behaviors of their second- and third-row congeners. However, the potential of first-row transition metals extends far beyond simply serving as cost-effective alternatives to noble metals. Their closed-shell reactivity is just the tip of the iceberg. These metals offer a rich manifold of reactivity patterns via open-shell configurations that could unlock a completely new dimension in organometallic chemistry—one that remains largely unexplored.

What direction do you think your research field should go in?

The future of transition metal catalysis should undoubtedly focus on advancing towards sustainability. This includes not only the development of earth-abundant first-row transition metal catalysts but also fostering a deeper understanding of reaction mechanisms at the molecular level. By doing so, we can optimize catalytic processes and design novel methodologies that significantly reduce waste and energy consumption—crucial goals for the ongoing drive to align scientific innovation with real-world applications. Not only that, I envision greater integration of multidisciplinary approaches, especially the application of machine learning and artificial intelligence, to accelerate the discovery of new reactions and address key challenges in synthetic chemistry. These emerging tools have the potential of helping us navigate the complexities of reaction pathways more efficiently, pushing the boundaries of what is possible in catalysis and facilitating breakthroughs that can meet the demands of both industry and society.

How would you describe your research philosophy?

My research group is focused on moving transition metal catalysis to a whole new level by giving reaction mechanism a novel dimension as a very powerful reaction design tool. Our goal is not only to streamline the development of more sustainable synthetic methodologies but also to unlock new reaction modes that are currently unprecedented in first-row transition metal catalysis. The foundation of our research program is to uncover the principles that govern catalytic transformations and propose tailored solutions to improve efficiency and create novel reactivity. Our distinctive approach includes trapping highly reactive reaction intermediates and using them as “knowledge building blocks” to identify potential bottlenecks within catalytic cycles. We place a strong emphasis on a multidisciplinary strategy, where catalysis, organometallic chemistry, organic synthesis, and cutting-edge techniques come together to provide a comprehensive understanding of reaction mechanisms. At the core of our philosophy are collaboration, creativity, and rigor. I believe that true breakthroughs in science occur when we push beyond traditional paradigms and boldly explore new, uncharted territories.

What aspects of your research do you find most exciting or most rewarding?

Without any doubt, working with my research group. I find it incredibly fulfilling to witness the personal and professional growth of my students. It is a true privilege to guide and mentor the next generation of scientists, helping them develop not only their technical expertise but also the leadership skills they need to excel in their careers. Watching them evolve into confident, independent researchers—and seeing their ideas contribute meaningfully to our work—gives me a great sense of accomplishment. Ultimately, the opportunity to help shape the future leaders of science is what makes my research so deeply meaningful and motivating every day.

Do you have any advice you would like to share with young researchers starting out in chemical research?

My advice to young researchers is rooted in two key principles: passion and resilience. First, follow your passion. It is the driving force that will not only keep you motivated during the inevitable challenges but also fuel the curiosity and creativity necessary for groundbreaking discoveries. Your passion will help you see possibilities where others may see barriers and will provide the energy needed to push the boundaries of knowledge.

Second, don’t fear failure. In research, setbacks are part of the journey. Embrace failure as a valuable learning experience, not a reason to quit. Each challenge offers insights that help you refine your approach and move closer to your goals. Be resilient and use every setback as an opportunity to grow—it’s often through failure that we make our most important breakthroughs. Stay persistent and remember that every failure brings you one step closer to the solution you’re seeking.

*This interview was conducted by the editors of Communications Chemistry*.

